# Relation between nutritional status on clinical outcomes of critically ill patients: emphasizing nutritional screening tools in a prospective cohort investigation

**DOI:** 10.1186/s40795-024-00869-3

**Published:** 2024-05-09

**Authors:** Omid Moradi Moghaddam, Masoumeh Hosseinzadeh Emam, Pardis Irandoost, Mahdi Hejazi, Zeinab Iraji, Leila Yazdanpanah, Seyedeh Farnaz Mirhosseini, Abolfazl Mollajan, Mohammad Niakan Lahiji

**Affiliations:** 1 Trauma and Injury Research Center, Iran University of Medical Sciences, Tehran, Iran; 2 Department of Critical Care, School of Medicine, Iran University of Medical Sciences, Tehran, Iran; 3 Department of clinical Nutrition, Rasool Akram Medical Complex Clinical Research Development Center, Iran University of Medical Sciences, Tehran, Iran; 4 Department of Biostatistics, Tarbiat Modares University, Tehran, Iran; 5 Department of Anesthesiology and Critical Care, Rasool-e-Akram Hospital, School of medicine, Iran University of Medical Sciences, Tehran, Iran

**Keywords:** Malnutrition, Intensive care unit, Nutritional risk assessment, Clinical outcome

## Abstract

**Background:**

Malnutrition is a significant concern reported in adult critically ill patients, yet there is no gold standard to assess nutritional status in this population. This study examines the association between nutritional status and clinical outcomes in intensive care unit (ICU) patients using nutritional risk assessment tools and aims to look for the best tool.

**Method:**

In a single-center prospective cohort study among 165 patients, the predictive performance of high or low malnutrition risk assessed by Nutritional Risk Screening (NRS), Modified Nutrition Risk in Critically Ill (m-NUTRIC), Mini-Nutritional-Assessment Short-Form (MNA-SF), Controlling Nutritional status (CONUT), and Prognostic Nutritional Index (PNI) were evaluated and compared for mortality, organ failure, length of hospitalization, and mechanical ventilation (MV).

**Results:**

Different assessment tools showed various nutritional statuses. m-NUTRIC and NRS-2002 were found to be associated more strongly relative to other tools with mortality (RR = 1.72; 95% CI, 1.42–2.08) and (RR = 1.37; 95% CI, 1.08–1.72), organ failure (RR = 1.69; 95% CI, 1.44–1.96) and (RR = 1.22; 95% CI, 0.99–1.48), MV (RR = 1.46; 95% CI, 1.27–1.65) and (RR = 1.21; 95% CI, 1.04–1.39) respectively. There was no correlation between malnutrition levels assessed by mentioned tools except for NRS-2002 and length of hospitalization. In predicting mortality or illness severity, the cut points were different for some tools like NUTRIC-score and all assessed outcomes (3.5), MNA-SF and mortality (6.5), CONUT with mortality, and MV (6.5).

**Conclusions:**

A considerable proportion of patients admitted to the ICU are at high risk for malnutrition. Compared to other tools, m-NUTRIC and NRS-2002 proved superior in predicting clinical outcomes in critically ill patients. Other tools overestimated the risk of malnutrition in the ICU so couldn’t predict clinical outcomes correctly.

**Supplementary Information:**

The online version contains supplementary material available at 10.1186/s40795-024-00869-3.

## Background

Malnutrition is a complex problem that is still largely unacknowledged. According to reports, malnutrition impacts 20–50% of acute care patients, with greater rates in intensive care [1]. Patients in intensive care units (ICU) are particularly vulnerable to malnutrition because their nutritional status worsens quickly, especially in the first week of critical illness [2]. Chronic and acute starvation, as well as the intensity of the underlying pathophysiological conditions that caused ICU admission, impact the nutritional status of ICU patients. As a result, within the first ten days of admission to the ICU, patients commonly lose between 5 and 25% of their lean body mass, depending on the severity of their organ failure. Earlier investigations demonstrate that patients with malnutrition are susceptible to worse outcomes such as longer hospital stay, higher incidence of overall complications, and mortality [3, 4]. Nutritional therapy can alleviate the consequences of malnutrition in critically ill patients [5]. Patients at risk of malnutrition or people with malnutrition must be identified so that appropriate nutritional support can be started in a timely manner [6]. As a result, patients’ nutritional status should be closely monitored, and proper nutritional support should be implemented as soon as possible to avoid negative consequences [7]. Malnutrition can be ministered by screening patients for nutritional risk using particular screening tools, followed by providing special nutritional treatment within 72 h of hospital admission [8, 9]. The soundest nutritional screening tool for patients is the one that best forecasts clinical outcomes during hospitalization [10]. Few investigations have examined the link between nutritional risk and clinical consequences in critically ill patients [1, 2, 10], and most of them had a retrospective design [2, 11]. The Academy of Nutrition and Dietetics recommends various combined methods due to the limitations of the available tools [12].

The aim of the study is to compare the performance of different nutrition screening tools in predicting negative clinical outcomes, such as mortality, mechanical ventilation (MV), multiple organ failure, and length of hospitalization in the ICU. The tools assessed were the five nutritional screening tools, including nutritional risk screening (NRS)-2002 [13], modified nutrition risk in the critically ill score (m-NUTRIC) [14], mini nutritional assessment short-form (MNA-SF) [15], controlling nutritional status (CONUT) [16], and prognostic nutritional index (PNI) [17]. These questionnaires have previously been used [18–23]; nevertheless, to the best of our knowledge, this study is the first comparison examination of these five questionnaires.

## Methods

### Study participant

A prospective cohort was performed in the Hazrat Rasool Akram Hospital, the main hospital center of the Iran University of Medical Sciences, with a 700-bed capacity. All patients who met the inclusion criteria and were hospitalized in the ICU of the university-associated educational hospital between September 1, 2021, and February 30, 2022, were included in this cohort study.

### Inclusion and exclusion criteria

The study comprised adult critically ill patients (≥ 18 years) who had spent at least three days in the ICU. In contrast, excluded were those who satisfied these criteria: [1] If the serum albumin, lymphocyte count, total cholesterol (TC), Body mass index (BMI) is missing; [2] patients were afflicted with hematological disorders; [3] patients who spent fewer than 72 h in the ICU [4] lack of information on m-NUTRIC parameters and other nutritional screening tools; [5] pregnancy and [6]. Patients who were re-admitted to the ICU from a general ward during the same hospital admission.

### Ethical approval

Iran University of Medical Sciences research ethics committee consented to all study procedures (IR.IUMS.FMD.REC.1400.466). The Helsinki Declaration principles were adhered to in their entirety. After thoroughly explaining the study’s objectives, informed permission was obtained from each patient or family member.

### Data extraction

The following parameters were chosen from the e-health record: Demographic characteristics (age, sex), clinical status (Acute Physiology and Chronic Health Evaluation II (APACHE-II), Sequential Organ Failure Assessment (SOFA), and Glasgow Coma Scale (GCS)), and laboratory data. weight (kg), was documented within the patient’s medical records, with an average estimation derived collaboratively by a nurse, nutritionist, and the examiner (a Critical Care Medicine Fellow). These estimations were estimated upon the patient’s dry weight and were subsequently adjusted to accommodate fluid retention, a process informed by consultations with both medical and nursing staff, alongside the examiner’s clinical evaluation for presence of edema, and height was measured based on the length of the ulna bone.

The SOFA score was used to assign patients to critical care units during the first 48 h of their stay and to determine the severity of their condition (discharge from the ICU or death). The APACHE-II score was calculated in patients admitted to the critical care unit within the first 24 h. Clinical outcomes and nutritional status, including occurrences of organ failure as assessed by the SOFA score (such as Respiratory, Coagulation, Hepatic, Cardiovascular, Renal, and CNS Failure), mortality rates, length of hospital stay, and utilization of nutritional support, were recorded.

At the start of each patient’s hospitalization, C-reactive protein (CRP), total lymphocyte count, CRP/Albumin, erythrocyte sedimentation rate (ESR), Total Cholesterol (TC), platelet, hematocrit, bilirubin, creatinine, sodium, potassium, arterial blood gas (ABG), and blood sugar (BS) were all assessed. mortality data were acquired from hospital records up to 28 days after ICU discharge.

### Nutritional screening

Following this, we used the NRS-2002, MNA-SF, m-NUTRIC, CONUT, and PNI to screen the nutritional status of all patients included in the study.

NRS-2002 was carried out by the European Society for Clinical Nutrition and Metabolism guidelines (ESPEN). Weight loss (%), reduced food intake (%), BMI (kg/m^2^), disease severity, disease type, and age are all included in this questionnaire. Patients are categorized as either no risk (NRS score ≤ 3) or at risk (NRS score > 3) [8].

Blood lymphocyte count and serum albumin concentration are used to generate the PNI. It was determined using the following formula:

“*10 × serum albumin (g/dl) + 0.005 × total lymphocyte count (*µL) “ [13].

Patients can be classified as malnourished (PNI value ≤ 38), and not malnourished (PNI value > 38) [2]. Higher scores on this questionnaire indicate a better nutritional status.

CONUT appears to be an effective tool for timely identification and ongoing control of hospital malnutrition, owing to its suitability for these screening activities. Serum albumin, lymphocyte counts, and TC levels were utilized to generate the CONUT score [14]. The CONUT has a point system of 12; 0 to 4 suggests normal nutritional status and 5 to 12 indicates malnutrition [15].

The MNA-SF was developed to diagnose malnutrition and assess the likelihood of malnutrition worsening. It has six sections to evaluate reduced food intakeand weight loss, activity, physical discomfort or acute illness, mental function, and BMI. This questionnaire has 14 points; a score of 12 to 14 indicates normal nutritional status, and a score of less than 12 demonstrates malnutrition [16]. Higher score underlines higher benefit from nutritional support and low score indicates low risk for complications. The m-NUTRIC score is the first tool developed and validated for adult critically ill patients [17]. Here, we employed the m-NUTRIC score (without interleukin 6), a nine-point scale derived from the NUTRIC score. Sum of points between 0 and 4 indicate that patients have a low malnutrition risk, sum between 5 and 9 are ‘high scores’ and are associated with worse clinical outcomes. We used the m-NUTRIC score, which ranges from 0 to 9 since IL-6 levels were not regularly evaluated in our chosen ICUs.

### Statistical analyses

Data were expressed as percentages for categorical, mean ±  standard deviation (SD) for numeric normal, and median (Interquartile range/IQR) for numeric non-normal variables. The relationship between nutritional status and clinical outcomes was investigated using logistic regression. The modeling strategy was backward, in which all the under-investigation variables and potential confounders (APACHE II, SOFA, length of ICU stay, BMI) were included in the model, and non-significant variables were removed from the model one by one. The nutritional status evaluated by different tools were entered as continuous variables (score) in regression models. The values of the odds ratio (OR) were obtained using adjusted logistic regression, and then risk ratio (RR) values were calculated using the following formula [18], in which P reference indicates the incidence of the outcome of interest in the nonexposed group:

Risk ratio= $$ \frac{OR}{(1-\text{p} \text{r}\text{e}\text{f}\text{e}\text{r}\text{e}\text{n}\text{c}\text{e})+(\text{p} \text{r}\text{e}\text{f}\text{e}\text{r}\text{e}\text{n}\text{c}\text{e} \times OR)}$$

The Spearman correlation coefficient calculated to evaluate the association between nutritional status score and APACHE II, SOFA, and length of ICU stay. Utilizing receiver operating characteristic (ROC) curves, nutritional risk tools were evaluated for their performance to predict mortality, organ failure, and MV. The area under the curve (AUC) analysis was used to examine the relevance of nutritional ratings in predicting clinical outcomes in the ICU. The statistical analysis of the data was performed using SPSS version 25 and *P* values < 0.05 were regarded as statistically significant.

## Results

**Fig. 1:**
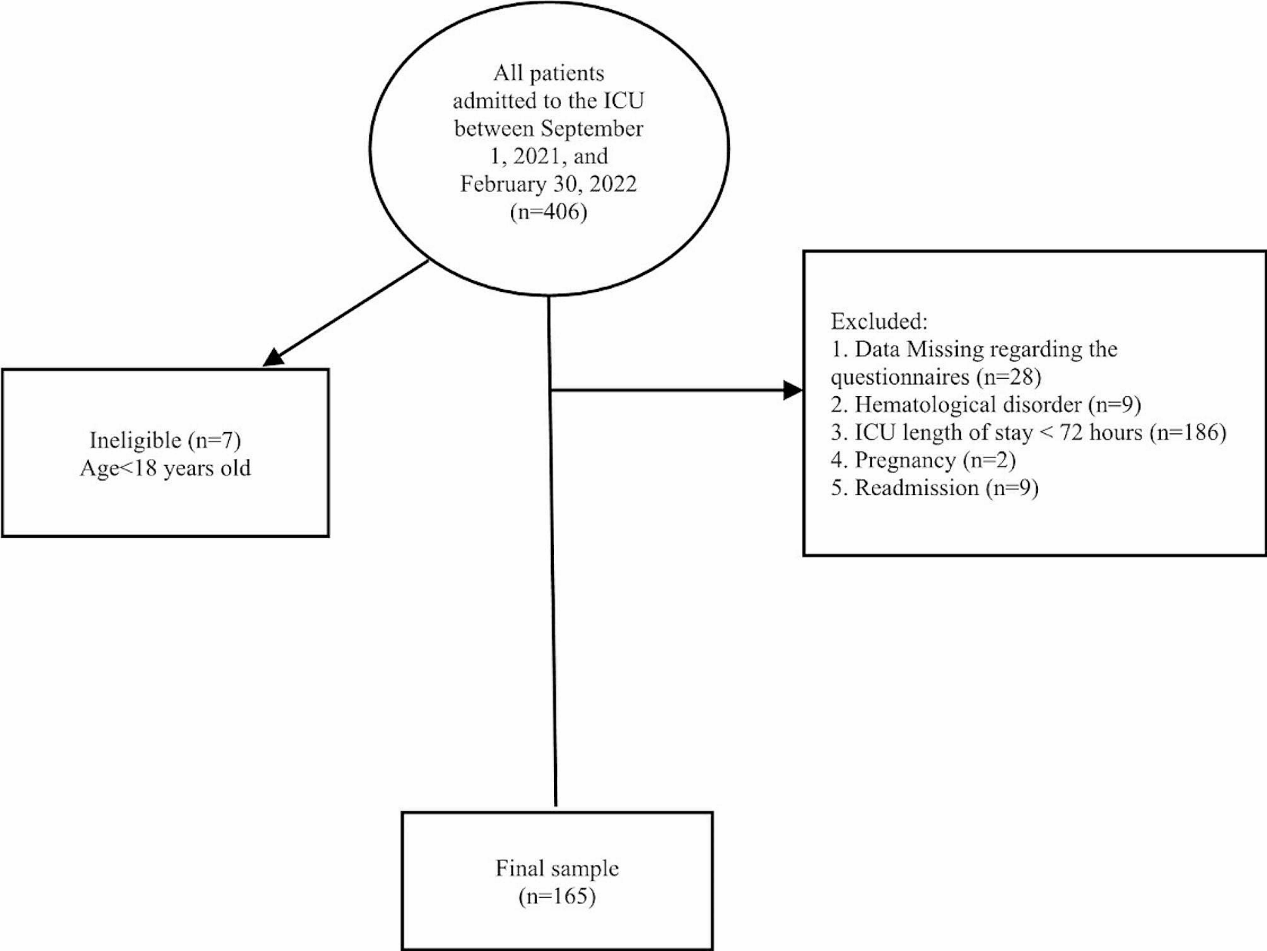
Flow diagram of the research design and patient enrollment in the analysis

**Fig. 2:**
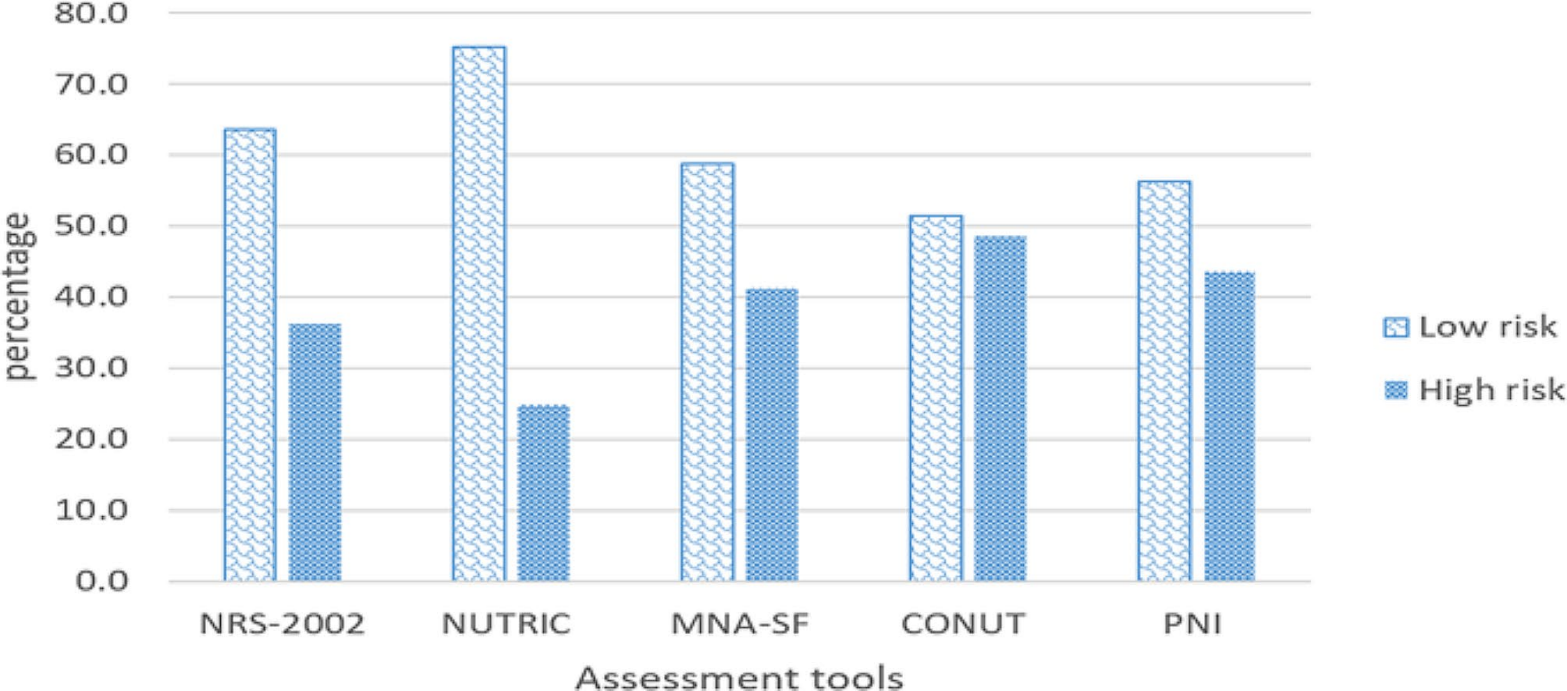
Percentage of malnourished patients assessed by different nutritional risk scores, NRS-2002: nutritional risk screening 2002; m-NUTRIC: modified nutrition risk in critically ill; MNA-SF: mini nutritional assessment-short form; PNI: prognostic nutrition index; CONUT: controlling nutritional status.

As shown in Fig. 1, a total of 165 patients were analyzed over the study period, as defined by inclusion and exclusion criteria. The most common comorbidities in patients were hypertension (*n* = 81, 49.1%), diabetes mellitus (*n* = 41, 24.8%), cardiovascular/vascular disease (*n* = 25, 15.2%), and cerebrovascular accident (*n* = 24, 14.5%). Furthermore, our study shows that 72 patients (44.2%) experienced organ failures, with the renal system (29.7%) and central nervous system (28.5%) exhibiting the highest incidence of observed organ failures, as determined by SOFA score assessment. Conversely, cardiovascular failures (5.5%) and respiratory failures (12.7%) were the least prevalent. Oral nutrition accounted for 32.7% of patients’ hospital nutrition, enteral nutrition provided 56.4%, and parenteral nutrition provided 10.9% of patients’ hospital nutrition. The MNA-SF, CONUT, m-NUTRIC, NRS-2002, and PNI tools were used to assess nutritional status (Fig. 2). According to our result, Using the CONUT, PNI, and MNA-SF questionnaires, a higher percentage of ICU patients were found to be at high risk for malnutrition (48.5%, 43.5%, and 41.2%, respectively). In contrast, NRS-2002 (36.3%) and the m-NUTRIC score (24/8%) showed the least proportion of patients having high risk.

Table 1 depicts the sample’s characterization based on the classifications of NRS-2002, m-NUTRIC, MNA-SF, CONUT, and PNI (low and high scores). The mean age and BMI of the patients in the entire sample were 59.32 ± 18.69 years and 25.82 ± 3.92, respectively, with 60.6%of patients (*n* = 100) being male. The mean SOFA and APACHE II scores were 5.37 ± 3.45 and 9.37 ± 6.49 respectively. Patients with higher NRS-2002, m-NUTRIC, and CONUT scores were older and had a higher APACHE II and SOFA score and lower GCSs (*P* < 0.05). Still, considering the MNA-SF and PNI grading systems, patients with lower scores were older and had a higher APACHE II and SOFA values and lower GCSs (P < 0.05). Based on the results of the m-NUTRIC and MNA-SF questionnaires, patients who were categorized as having a high or low risk of malnutrition had significantly different BMI range.

**Table 1:** Patient features according to nutritional risk screening tools

Variable		Age	GCS	SOFA score	APACHE II score	BMI
All sample		59.32 ± 18.69	11.11 ± 3.90	5.37 ± 3.45	9.37 ± 6.49	25.82 ± 3.92
**NRS-2002** **<3 vs. ≤ 3**	Low	53.60 ± 17.60	12.69 ± 3.21	3.81 ± 2.30	6.70 ± 4.60	25.94 ± 3.71
	High	69.33 ± 16.29	8.35 ± 3.45	8.08 ± 3.46	14.03 ± 6.72	25.60 ± 4.30
	*P value*	*< 0.001*	*< 0.001*	*< 0.001*	*< 0.001*	*0.616*
**m-NUTRIC** **≥5 Vs < 5**	Low	55.50 ± 17.47	12.45 ± 3.33	4.08 ± 2.23	7.04 ± 4.24	26.26 ± 3.82
	High	70.90 ± 17.65	7.07 ± 2.46	9.26 ± 3.57	16.41 ± 7.06	24.48 ± 3.96
	*P value*	*< 0.001*	*< 0.001*	*< 0.001*	*< 0.001*	*0.015*
**MNA-SF** **< 12 Vs ≥ 12**	Low	62.91 ± 19.02	10.39 ± 3.94	6.61 ± 3.66	11.06 ± 7.42	24.72 ± 4.09
	High	56.81 ± 18.14	11.61 ± 3.81	4.49 ± 3.01	8.18 ± 5.49	26.58 ± 3.63
	*P value*	*0.041*	*0.049*	*< 0.001*	*0.010*	*0.003*
**CONUT** **> 4 Vs ≤ 4**	Low	55.99 ± 18.26	12.43 ± 3.24	4.13 ± 2.41	7.85 ± 5.28	25.86 ± 3.59
	High	62.87 ± 18.61	9.71 ± 4.07	6.69 ± 3.87	10.99 ± 7.26	25.77 ± 4.27
	*P value*	*0.018*	*< 0.001*	*< 0.001*	*0.004*	*0.884*
**PNI** **≤ 38Vs > 38**	low	64.72 ± 18.56	9.76 ± 4.20	6.65 ± 4.21	11.05 ± 7.58	25.57 ± 3.77
	high	55.15 ± 17.81	12.16 ± 3.32	4.38 ± 2.30	8.06 ± 5.19	26.01 ± 4.05
	*P value*	*0.003*	*< 0.001*	*< 0.001*	*0.017*	*0.466*

BMI: body mass index; APACHE: acute physiology and chronic health evaluation; SOFA: sequential organ failure assessment; NRS: nutritional risk screening; m-NUTRIC: modified nutrition risk in critically ill; MNA-SF: mini nutritional assessment short-form; CONUT: controlling nutritional status; PNI: prognostic nutritional index. Data are presented as Mean ± SD. *P* < 0.05 was considered statistically significant

Table 2 displays the relative risks for outcomes based on the NRS-2002, m-NUTRIC, MNA-SF, CONUT, and PNI. On report to the logistic model, m-NUTRIC was found to be associated with mortality (RR: 1.72; 95% CI, 1.42–2.08; *P* < 0.001), organ failure (RR: 1.69; 95% CI, 1.44–1.96; *P* < 0.001), and MV (RR: 1.46; 95% CI, 1.27–1.65; *P* < 0.001). In contrast, the score of malnutrition as measured by MNA-SF had no significant correlation with mortality, organ failure, and MV. In comparison, NRS-2002 and PNI have significant correlations with mortality (RR: 1.37; 95% CI, 1.08– 1.72; *P* = 0.011), (RR: 0.94; 95% CI, 0.90– 0.98; *P* = 0.007) and MV, respectively (RR: 1.21; 95% CI, 1.04– 1.39; *P* = 0.015), (RR: 0.97; 95% CI, 0.95– 0.99; *P* = 0.024). The relative risk for nutritional score and mortality, organ failure, and MV were depicted as Fig. S1.

**Table 2:** Relationship between nutritional score and mortality, organ failure, and MV

Nutritional screening tool	Mortality RR (95% CI)	*P*-value	Organ failure RR (95% CI)	*P*-value	MV RR (95% CI)	*P*-value
**NRS-2002**	1.37 (1.08–1.72)	0.011	1.22 (0.99– 1.48)	0.066	1.21 (1.04–1.39)	0.015
**NUTRIC**	1.72 (1.42–2.08)	< 0.001	1.69 (1.44–1.96)	< 0.001	1.46 (1.27–1.65)	< 0.001
**MNA-SF**	1.04 (0.94–1.15)	0.418	0.96 (0.89– 1.04)	0.355	1.00 (0.95–1.06)	0.849
**CONUT**	1.17 (1.00-1.37)	0.048	1.21 (1.04– 1.39)	0.013	1.02 (0.89–1.11)	0.733
**PNI**	0.94 (0.90–0.98)	0.007	0.99 (0.96–1.03)	0.769	0.97 (0.95–0.99)	0.024

NRS: nutritional risk screening; m-NUTRIC: modified nutrition risk in critically ill; MNA-SF: mini nutritional assessment short-form; CONUT: controlling nutritional status; PNI: prognostic nutritional index. *P* < 0.05 was considered statistically significant

The relationship between quantitative data including length of ICU stay, APACHE II, and SOFA scores, with nutritional scores based on different questionnaires, was expressed using a correlation in Table 3. Except for the NRS-2002, no statistically significant association between nutritional scores and length of stay in the ICU has been shown. However, there was a significant correlation between CONUT and APACHE II and SOFA scores. CONUT scores correlated positively with the APACHE II and SOFA, and APACHE II and SOFA scores rise with an increase in the questionnaire score indicating a shift toward malnutrition. MNA-SF and PNI, on the other hand, had a negative correlation and APACHE II and SOFA scores rise with a decrease in the questionnaire score indicating a shift toward malnutrition. As shown in Table 3, The strongest correlation between mentioned assessment tools and APACHE II and SOFA scores was for NRS-2002 and m-NUTRIC questionnaires.

**Table 3:** Correlation of nutritional scores of patients with a length of hospitalization, APACHE II, and SOFA scores

Nutritional screening tool	Length of ICU Stay		APACHE II score		SOFA score	
	Correlation	*P*-value	Correlation	*P*-value	Correlation	*P*-value
NRS-2002	0.201	0.010	0.566	< 0.001	0.692	< 0.001
m-NUTRIC	0.137	0.081	0.612	< 0.001	0.646	< 0.001
MNA-SF	− 0.119	0.130	− 0.315	< 0.001	− 0.461	< 0.001
CONUT	0.109	0.164	0.326	< 0.001	0.383	< 0.001
PNI	− 0.037	0.639	− 0.258	0.001	− 0.337	< 0.001

NRS: nutritional risk screening; m-NUTRIC: modified nutrition risk in critically ill; MNA-SF: mini nutritional assessment short-form; CONUT: controlling nutritional status; PNI: prognostic nutritional index. *P* < 0.05 was considered statistically significant

**Fig. 3:**
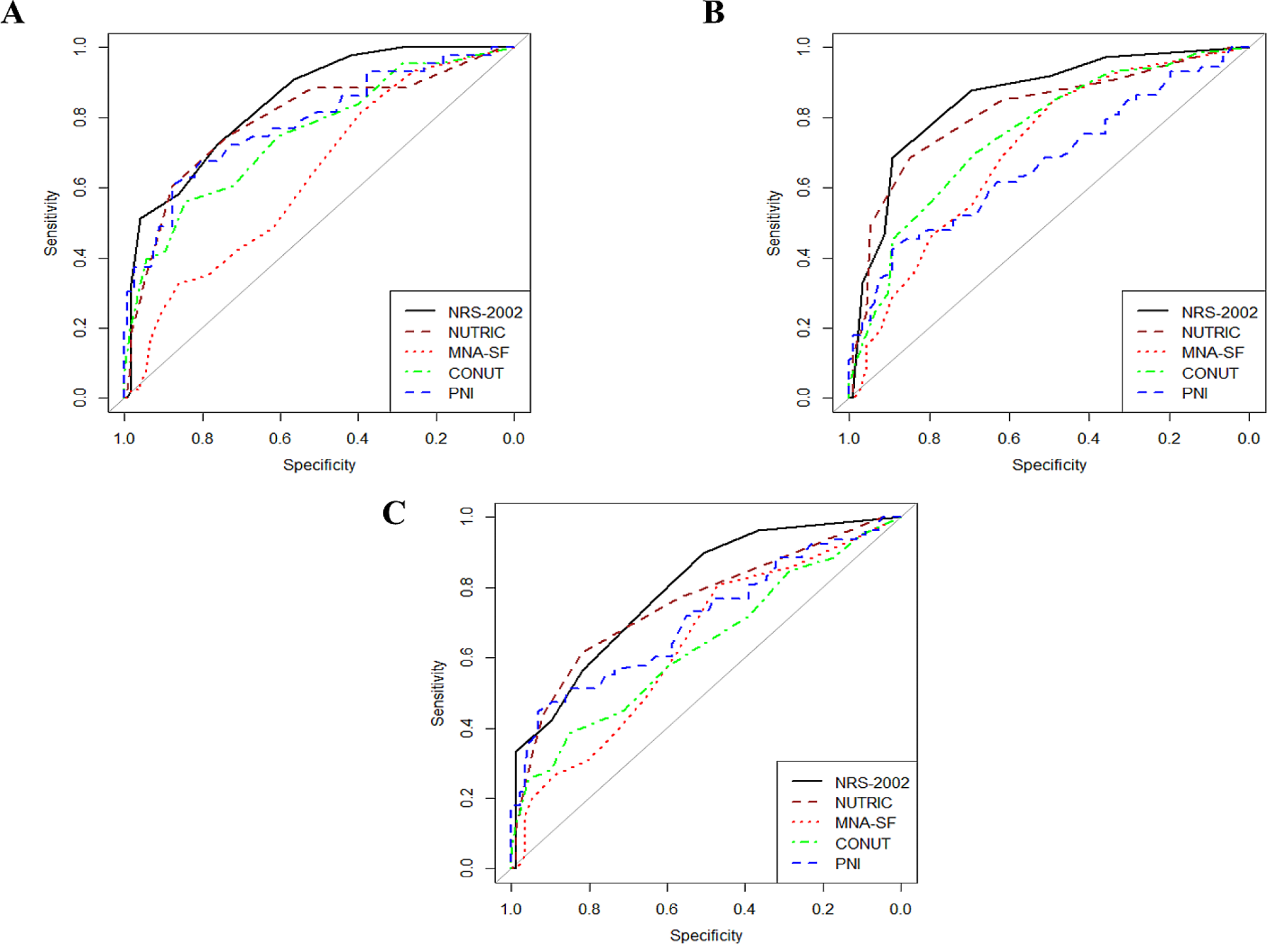
ROC curve for nutritional risk assessment tools for predicting mortality, organ failure, and MV. ROC: Receiver operating characteristic; NRS-2002: nutritional risk screening 2002; m-NUTRIC: modified nutrition risk in critically ill; MNA-SF: mini nutritional assessment-short form; PNI: prognostic nutrition index; CONUT: controlling nutritional status, **A**: Nutritional score for predicting mortality, **B**: Nutritional score for predicting organ failure, **C**: Nutritional score for predicting MV

Table 4 demonstrates the overall performance of nutritional scores for the prediction mortality, organ failure, and MV. The ROC curves of NRS-2002, m-NUTRIC, MNA-SF, CONUT, and PNI revealed that the values in predicting mortality, organ failure, and MV were the highest for NRS-2002 and m-NUTRIC scores. The corresponding cutoff score for NUTRIC was 3.5 for all mentioned outcomes but it was different in the case of NRS-2002 for MV in comparison to other assessed outcomes (1.5 vs. 3.5). In the current study, MNA-SF had different cutoff points for mortality relative to organ failure and MV (6.5 vs. 12.5). Moreover, the cutoff point of organ failure in the case of CONUT was lower than mortality and MV (4.5 vs. 6.5). PNI had the same cutoff point in our study for all of the outcomes (34.75). In Fig. 3, ROC curves illustrate the nutritional tools’ ability to predict mortality and organ failure, and MV. The ROC curve was developed using each tool’s predictive ability by its AUC.

**Table 4:** The value of nutrition scores in predicting mortality, organ failure, and MV

Nutritional screening tool		NRS-2002	NUTRIC	MNA-SF	CONUT	PNI
Mortality	AUC (95% CI)	0.844 (0.78-90)	0.791 (0.70–87)	0.631 (0.53–0.72)	0.750 (0.66–0.83)	0.785 (0.70–0.87)
	Cut-off point	3.5	3.5	6.5	6.5	34.75
	Sensitivity	0.721	0.744	0.910	0.558	0.877
	Specificity	0.762	0.738	0.233	0.844	0.605
Organ failure	AUC (95% CI)	0.854 (0.79–0.91)	0.814 (0.74–0.88)	0.707 (0.62–0.78)	0.754	0.670 (0.58–0.75)
	Cut-off point	3.5	3.5	12.5	4.5	34.75
	Sensitivity	0.685	0.685	0.489	0.699	0.891
	Specificity	0.891	0.848	0.849	0.685	0.425
MV	AUC (95% CI)	0.791 (0.72–0.85)	0.754 (0.67–0.82)	0.637 (0.55–0.72)	0.633 (0.54–0.71)	0.707 (0.62–0.78)
	Cut-off point	1.5	3.5	12.5	6.5	34.75
	Sensitivity	0.897	0.615	0.471	0.385	0.931
	Specificity	0.506	0.816	0.808	0.851	0.449

AUC: Area under the ROC curve; CI: Confidence interval; NRS: nutritional risk screening; m-NUTRIC: modified nutrition risk in critically ill; MNA-SF: mini nutritional assessment short-form; CONUT: controlling nutritional status; PNI: prognostic nutritional index, ROC: receiver operating characteristic

## Discussion

The current study may be the first to report an association between five nutritional screening tools and APACHE II, SOFA scores as well as clinical outcomes (mortality, MV, organ failure, and length of ICU hospitalization) in Iranian ICU patients. In the first phase of the study, we estimated the prevalence of malnutrition separately for each questionnaire. Malnutrition worsens clinical outcomes via multiple mechanisms, including systemic inflammation, diminished immune function, and mitochondrial dysfunction [19]. So, we explored the association between each questionnaire and clinical outcomes in the second phase of our study. Our results demonstrated that the nutritional risk findings from the five screening tools varied. The prevalence of malnutrition was calculated as follows, descending from highest to lowest: CONUT (48.5%), PNI (43.6%), MNA-SF (41.2%), NRS-2002 (36.3%), and m-NUTRIC (24.8%). Various questionnaires are looked into the prevalence of malnutrition in critically ill patients in different studies [20–22]. To the best of our knowledge, there has not been a study that evaluated and compared five questionnaires simultaneously to examine the incidence of malnutrition; however, different studies find varying malnutrition statuses based on their nutritional screening tools [23–26]. Clinical condition, nutritional screening tool, disease type/severity, and research methodology all explain these variations. Understanding nutritional characteristics and their predictive importance are challenging [27]. Also, defining which assessing tools and cutoff point has the best correlation with clinical outcomes is a highly prominent factor. Therefore, we assessed the best cutoff points in predicting different clinical outcomes for mentioned nutritional tools.

In our study, it is essential to state that only NRS-2002 significantly correlated with the ICU hospitalization duration. Also, m-NUTRIC score showed correlation with duration of ICU hospitalization but not in significant levels. In some previous studies, nutritional screening tools predicted length of hospitalization [1, 7, 28]. A study conducted on 440 patients admitted to the ICU found that m-NUTRIC was correlated with length of stay [29]. In a large study involving 987 elderly ICU patients in Albania, NRS-2002 was found to be significantly associated with 28-day mortality and length of stay, but not with MV days [30]. Some studies on various clinical situations, including hip fracture, surgery, hepatocellular carcinoma, colorectal cancer, and esophageal cancer, have demonstrated the ability of MNA-SF, CONUT, and PNI to predict the length of hospitalization [31–37]. However, the predictive ability of these tools concerning the length of ICU hospitalization for patients has not been thoroughly examined. The lack of correlation is since the relationship between hospitalization stay, and nutritional status is not always a cause-and-effect connection. In current investigation, MNA-SF did not show a statistically significant relationship between nutritional scores and mortality, prolonged MV, organ failure, or length of hospital stay; this suggests that the MNA-SF was not appropriate questionnaire for estimating malnutrition and malnutrition-related complications. However, MNA-SF questionnaire has a significant correlation with APACHE II and SOFA scores. The MNA-SF score has been developed to explore malnutrition among the elderly population [16]. Therefore, in its scoring system, there is a focus on conditions (like cognitive function and mobility) that are more relevant to this age group and not necessarily all hospitalized or critically ill patients. It may explain why MNA-SF showed a poor predictive ability for clinical outcomes in the ICU setting.

PNI score had only association with mortality and MV. PNI score is calculated using two variables: serum albumin and total lymphocyte count. Although serum albumin is associated with mortality risk, it has shown poor sensitivity and specificity for clinical outcomes prediction in critically ill patients [38]. Therefore, the PNI has been commonly used in patients with coronary artery disease and malignancy [39–41]; and its accuracy may not be adequate for ICU patients.

In our study, while CONUT identified more individuals at high risk of malnutrition compared to other assessment tools, it only revealed statistically significant associations with organ failure. Also, the association of nutritional score based on CONUT with mortality was marginally significant. The CONUT score’s limited ability to predict clinical outcomes in ICU patients may be attributed to the limitations of serum albumin, a factor used in its calculation, especially given its low predictive value in critical illness patients. Our findings suggest that CONUT overestimates the malnutrition in critically ill patients. A previous survey of 461 diabetic patients using the CONUT screening tool revealed that 38% of patients were malnourished [42]. The results of present study are consistent with a recent observational study conducted on 365 hospitalized patients, which revealed the highest prevalence of malnutrition screening determined by CONUT [43]. Although NRS 2002 and CONUT detected different levels of nutritional risk (CONUT: 48.5%; NRS-2002: 37% ), they were similar in their ability to foresee organ failure. All used tools had a significant relation with organ failure but no other outcomes. Given these, NRS-2002 and m-NUTRIC scores outperform CONUT in predicting malnutrition and malnutrition-related complications in critically ill patients. In current study, m-NUTRIC score was significantly related to mortality, organ failure, and MV duration. This tool is also highly correlated with APACHE II and SOFA scores. It is noteworthy to mention that the observed correlation might result from the inclusion of APACHE II and SOFA scores in the m-NUTRIC questionnaire. Furthermore, the m-NUTRIC score encompasses three additional components, including age, number of comorbidities, and days from hospital to ICU admission, all of which could have an impact on this correlation.

In several studies, nutritional status, as measured by the m-NUTRIC score, is correlated with an increased risk of mortality, organ failure, and the need for prolonged MV [11, 44–49].

Despite estimating a lower prevalence of people at high risk of malnutrition than other questionnaires, m-NUTRIC and NRS-2002 have shown superior performance in predicting clinical outcomes (mortality, organ failure, prolonged MV, APACHE II and SOFA scores). As a result, we can suggest that the NRS-2002 and m-NUTRIC tool evaluate clinical outcomes better than other screening tools in patients hospitalized in the ICU. Also, patients with a higher NRS-2002 and m-NUTRIC score require further investigations for nutritional treatment. m-NUTRIC was created specifically for critically ill patients. It can also be used quickly and efficiently when patients cannot communicate. On the other hand, compared to other screening questionnaires, more criteria have been considered for patients’ clinical conditions [17]. However, this tool has a few limitations that need to be regarded. For example, no nutrition parameters are included in m-NUTRIC; it could be the reason for the lower malnutrition rate identified by m-NUTRIC compared to other questionnaires in our study. Designing questionnaire with m-NUTRIC parameters along with some nutritional parameters could be suggested in future studies for finding malnourished patients at risk of malnutrition-related complications.

Other than the m-NUTRIC and NRS-2002 questionnaires, none of the tools could accurately foretell the outcomes. Our research reveals that the three tools, PNI, CONUT, and MNA, have various cutoff values, which could be related to multiple factors. It is possible that different cutoff points of screening tools can be considered for predicting different outcomes. Further research with larger sample sizes is recommended for assessing the best cutoff points for ICU relate outcomes. The findings suggest that some assessment methods are more accurate in diagnosing specific clinical outcomes than others. As a result, future research should assess each outcome separately using different questionnaires to assure accuracy. Our study has some limitations that should be noted. First, anthropometric measurements were estimated, which increases the risk of mistakes. Second, only one evaluation of five nutritional tools was conducted upon admission. Our study did not consider changes occurring in nutritional markers, which may be greater predictors of unfavorable outcomes. Finally, due to the small sample size, we could not undertake subgroup analyses to determine the optimum malnutrition screening technique for each disease specifically.

## Conclusions

In conclusion, our research shows that the mortality rates of patients in the ICU are substantially connected with malnutrition on admission. We propose using these simple nutritional ratings to identify patients at nutritional risk, since they help lead the creation of effective and timely intervention approaches. Because of the specificity of this tool in critical care patients, it appears that the m-NUTRIC performs better in predicting clinical outcomes. Also, The NRS-2002 score has more sensitivity than the other nutritional screening tools in predicting mortality and organ failure.

### Electronic Supplementary Material

Below is the link to the electronic supplementary material.

Supplementary Material 1

## Data Availability

All data generated or analysed during this study are included in this published article [and its supplementary information files]. Also, for further information please contact with following Email address: Pardis.id@gmail.com.

## Abbreviations

APACHE: Physiology and Chronic Health Evaluation II
AUC: Area under the ROC curve
BMI: Body mass index
COUNT: Controlling Nutritional status
ESPEN: European Society of Parenteral and Enteral Nutrition guidelines
GCS: Glasgow Coma Scale
IQR: Interquartile range
ICU: Intensive care unit
MV: Mechanical ventilation
MNA-SF: Mini-Nutritional-Assessment Short-Form
m-NUTRIC: Modified Nutrition Risk in Critically Ill
NRS: Nutritional Risk Screening
PNI: Prognostic Nutritional Index
SOFA: Sequential Organ Failure Assessment
SD: Standard deviation
TC: Total cholesterol
