# Supplementary material for: Relation between nutritional status on clinical outcomes of critically ill patients: emphasizing nutritional screening tools in a prospective cohort investigation
Source: BMC Nutr. 2024 May 9;10:69. doi: 10.1186/s40795-024-00869-3 (PMC11080301; doi:10.1186/s40795-024-00869-3)
Supplement: Supplementary file 1 — Supplementary Material 1 [file 40795_2024_869_MOESM1_ESM.docx]

NRS-2002: nutritional risk screening 2002; m-NUTRIC: modified nutrition risk in critically ill; MNA-SF: mini nutritional assessment-short form; PNI: prognostic nutrition index. Screening tests were significant (P < 0.05).

**Figure S1**: Relative Risk for nutritional score and Mortality, Organ failure, and Mechanical ventilation
